# Prevalence and Incidence of Huntington's Disease: An Updated Systematic Review and Meta‐Analysis

**DOI:** 10.1002/mds.29228

**Published:** 2022-09-26

**Authors:** Alex Medina, Yasamin Mahjoub, Larry Shaver, Tamara Pringsheim

**Affiliations:** ^1^ Department of Clinical Neurosciences, Cumming School of Medicine University of Calgary Calgary Alberta Canada; ^2^ Adult Chronic Diseases and Conditions Division Public Health Agency of Canada Nepean Ontario Canada; ^3^ Hotchkiss Brain Institute University of Calgary Calgary Alberta Canada; ^4^ Department of Psychiatry, Pediatrics, Community Health Sciences University of Calgary Calgary Alberta Canada

**Keywords:** Huntington's disease, incidence studies, prevalence studies

## Abstract

The incidence and prevalence of Huntington's disease (HD) based on a systematic review and meta‐analysis of 20 studies published from 1985 to 2010 was estimated at 0.38 per 100,000 person‐years (95% confidence interval [CI], 0.16–0.94) and 2.71 per 100,000 persons (95% CI, 1.55–4.72), respectively. Since 2010, there have been many new epidemiological studies of HD. We sought to update the global estimates of HD incidence and prevalence using data published up to February 2022 and perform additional analyses based on study continent. Medline and Embase were searched for epidemiological studies of HD published between 2010 and 2022. Risk of bias was assessed using a quality assessment tool. Estimated pooled prevalence or incidence was calculated using a random‐effects meta‐analysis. A total of 33 studies published between 2010 and 2022 were included. Pooled incidence was 0.48 cases per 100,000 person‐years (95% CI, 0.33–0.63). Subgroup analysis by continent demonstrated a significantly higher incidence of HD in Europe and North America than in Asia. Pooled prevalence was 4.88 per 100,000 (95% CI, 3.38–7.06). Subanalyses by continent demonstrated that the prevalence of HD was significantly higher in Europe and North America than in Africa. The minor increase in prevalence (more so than incidence) demonstrated in this updated review could relate to the enhanced availability of molecular testing, earlier diagnosis, increased life expectancy, and de novo mutations. Limitations include variable case ascertainment methods and lacking case validation data. © 2022 Her Majesty the Queen in Right of Canada. *Movement Disorders* published by Wiley Periodicals LLC on behalf of International Parkinson and Movement Disorder Society. Reproduced with the permission of the Minister of Public Health Agency of Canada.

## Graphical abstract

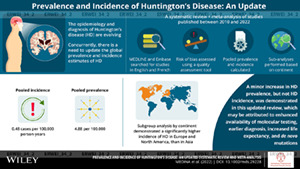

December Infographic: Prevalence and Incidence of Huntington's Disease: An Updated Systematic Review and Meta‐Analysis

Huntington's disease (HD) is a neurodegenerative condition with a wide neuropsychiatric clinical spectrum that may involve different combinations of movement disorders (primarily chorea), dementia, and behavioral or psychiatric manifestations.^1^ HD is a polyglutamine disease caused by a CAG trinucleotide repeat expansion in the huntingtin gene (*HTT*), located on chromosome 4. It is inherited in an autosomal dominant pattern. The normal repeat range length of CAG lies between 10 to 35. There is a low penetrance range from 36 to 39 repeats, whereas patients with 40 or more repeats will most likely express the condition.^1^ In addition, there is a strong inverse correlation between repeat length and age of onset.^2^

HD is a clinical diagnosis made through the evaluation of family history, personal history, neurological and psychiatric examination, and genetic testing. Current classification schemes subdivide HD into presymptomatic, prodromal, and manifest HD.^3^ Presymptomatic HD includes individuals who have the CAG expansion but currently have no signs or symptoms related to HD. Prodromal HD includes individuals with the CAG expansion who have nonspecific or possible motor abnormalities on exam and subtle but clear cognitive changes. Manifest HD includes individuals with the CAG expansion with greater than 90% confidence of motor abnormalities plus minor or major neurocognitive changes or individuals with greater than 99% probability of motor abnormalities with unchanged cognition. Individuals who have not had genetic testing for HD but in whom this diagnosis is suspected on clinical grounds would be categorized similarly as the following: at risk for HD, but not manifest; clinically prodromal HD; and clinically manifest HD.

The pooled incidence of HD based on studies published from 1985 to 2010 has been estimated as 0.38 per 100,000 person‐years (95% confidence interval [CI], 0.16–0.94), with a global overall prevalence of 2.71 per 100,000 (95% CI, 1.55–4.72).^4^ More recent studies highlight that there is an increment in prevalence in some regions.^5^, ^6^, ^7^, ^8^, ^9^, ^10^, ^11^, ^12^ Although there are plausible explanations for variations and increased rates across populations, such as the identification of different *HTT* gene haplotypes, health care accessibility, attitudes that differ on illness‐related stigma, migration, and the identification of HD cluster regions, specific determinants remain to be elucidated.

Variability in case ascertainment may play a role in the regional heterogeneity of reported epidemiological data. Prior to the availability of genetic testing in the mid‐1990s, epidemiological studies relied primarily on the motor clinical spectrum along with a positive family history of similar manifestations. This approach could have overestimated the actual rates by including HD phenocopies. On the other hand, de novo gene mutations, which have been estimated to represent up to 7.1% of the cases,^13^ were likely neglected because of the absence of a clear transmission pattern. The identification, mapping, and subsequent cloning of the *HTT* gene facilitated a molecular diagnosis, permitted predictive testing, and likely increased the yield of detection in atypical forms of presentation. In addition, at‐risk population and uptake of predictive testing calculations surfaced as measures to identify cases for clinical practice as well as for research purposes. However, the initial availability of molecular testing was limited, even in jurisdictions with robust health care systems, and the diagnostic inclusion criteria for epidemiological studies largely persisted under solely clinical grounds. Since the previously published systematic review and meta‐analysis on the global prevalence and incidence of HD, there have been many new epidemiological reports in the literature (particularly since 2016), with expanded utilization of confirmatory genetic testing for case ascertainment.

Therefore, given the evolving landscape of HD epidemiology and diagnosis, in this study we aimed to update the global prevalence and incidence estimates of HD using epidemiological data published since 2010.

## 1 Methods

A systematic review and meta‐analysis were performed following a predetermined protocol submitted and registered with PROSPERO (CRD42021234714). The protocol followed the standards recommended in the Preferred Reporting Items for Systematic Reviews and Meta‐Analysis protocols.^14^

### 1.1 Search Strategy and Literature Source

The literature search was developed, peer reviewed by a health science librarian, and applied to two electronic databases (MEDLINE and EMBASE). The initial search was limited to studies from January 2010 to March 2021 (with an updated search in February 2022); the search was restricted to studies in English and French. We intentionally overlapped the search by 1 year with our previous systematic review; studies reported in the previously published 2012 review^4^ were excluded. Controlled vocabulary was used for HD, prevalence, and incidence. In addition, the text word and key word terms were added to broaden our search to incorporate preindexing studies. The terms were subsequently combined with “or” obtaining term clusters, which were then combined with “and.” The articles derived from each database were combined in Endnote X9 (Clarivate, UK) and subsequently uploaded to the COVIDENCE platform v2536 8 cc39587 (Australia) for duplicate removal, screening, and extraction stages. Review articles and book chapters on the topic were additionally explored to identify studies not captured through our search. The complete search strategy parameters for the two databases can be found in Appendix S1.

### 1.2 Study Screening and Selection

Citations and abstracts examining the prevalence or incidence of HD were included and evaluated independently through COVIDENCE platforms by two authors (A.M., T.P.). Title and abstract screening were conducted to determine if the study examined the prevalence or incidence of HD in a specific geographical region or population, irrespective of the ascertainment method used. If the study met the inclusion criteria, it was selected and uploaded for the full‐text review stage; discordant selections were evaluated a second time to reconcile for inclusion or exclusion. The citations selected for full‐text review were filtered based on the following exclusion criteria: conference abstract, prevalence or incidence not reported, editorial or book chapter, duplicate, article not in English or French, article included in previous review, or same data published in another study. Interrater reliability was calculated in COVIDENCE using Cohen's κ statistic.

### 1.3 Data Extraction from Selected Studies

Data extraction was performed using a standardized data collection table, one for incidence and one for prevalence studies by two authors (A.M., T.P.). The tables were reviewed for the accuracy of the information by a third author (Y.M.). The variables included were first author, year of publication, country, population size, number of cases, the data source used to identify cases, diagnostic criteria used, incidence/prevalence date, calculated incidence/prevalence, and subgroup calculated incidence or prevalence. We only included unique, nonoverlapping samples to avoid multiple publication biases. After a cross‐check process, a consolidated entry form was subsequently developed.

### 1.4 Risk of Bias

A quality assessment was performed for each study included. Each study was given a score out of four based on (1) definition of the study population, (2) standardization of data collection methods, (3) use of valid clinical criteria and/or genetic testing to assess for the presence of disease, and (4) incidence and/or prevalence reporting with CIs and by subgroup.

### 1.5 Data Synthesis and Analyses

The included studies were classified according to whether they examined the prevalence or incidence of HD. Subsequently, an estimated pooled prevalence or incidence was calculated using Comprehensive Meta‐Analysis Software version 3.3.070 (USA). The raw data, including population counts and number of cases, for both prevalence and incidence studies were used to calculate the individual study prevalence or incidence rates, corroborated by checking the estimates provided in each study. For incidence estimates, where incidence was provided per year or per group of years, the most recent incidence was used in calculations to reflect the latest data. If studies did not report incidence rates for each year, the average incidence was used in the meta‐analysis. For both prevalence and incidence data, the number of cases or population size was back calculated when necessary (if either were not reported in the original publication). We report the prevalence and incidence of HD based on studies published between 2011 and 2021. We also combined these studies with the those included in the previously published 2012 review^4^ to calculate estimates based on all studies published from 1985 to 2021. Heterogeneity in pooled estimates was reported using the *I* ^2^ statistic and the *Q* statistic. We combined the studies using a random‐effects meta‐analysis. We performed subanalyses based on the continent where the study was performed (Africa, Asia, Europe, North America, South America, and Oceania) when possible. A post hoc sensitivity analyses was performed in which the 2011 to 2021 analysis was repeated, eliminating studies that did not use valid clinical criteria or genetic testing to assess for the presence or absence of disease (ie, studies using administrative data with no case validation procedure).

**FIG 1:**
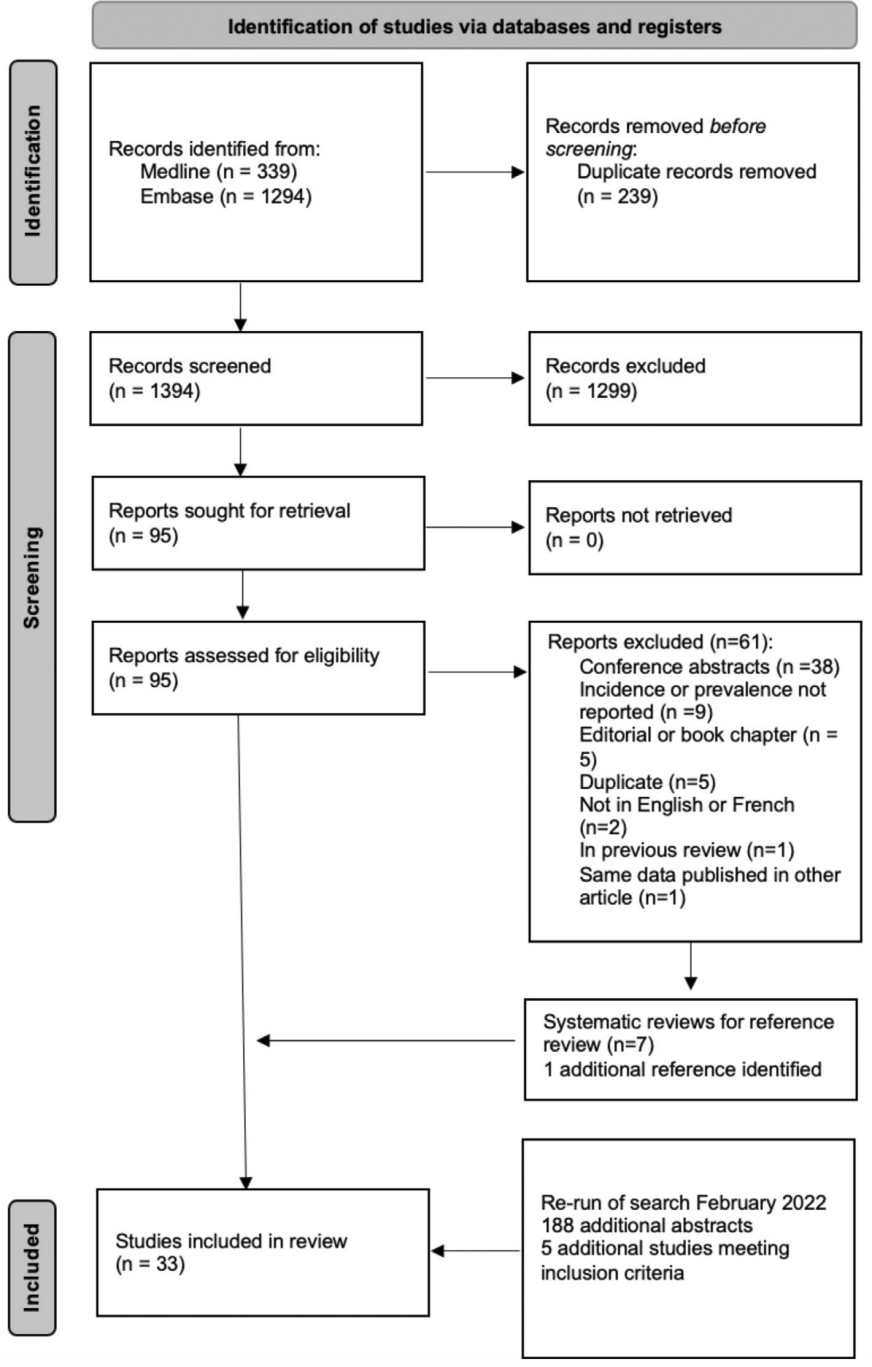
Preferred Reporting Items for Systematic Reviews and Meta‐Analyses 2020 flow diagram for new systematic reviews, which included searches of databases and registers only.

## 2 Results

The electronic database search from 2010 to March 2021 identified 1633 citations, 339 in MEDLINE and 1294 in EMBASE. A total of 239 duplicates were removed, resulting in 1394 studies (Fig. 1). In the abstract screening stage, 1299 irrelevant citations were excluded. We examined the full text of 95 studies and excluded another 61. The main reasons for exclusion at this stage were the following: conference abstracts (n = 38), outcomes of interest not discussed (n = 9), editorial or book chapters (n = 5), duplicates not filtered through COVIDENCE (n = 5), articles not written in English or French (n = 2), article included in previous review (n = 1), and publication using the same data in another included study (n = 1). Seven review articles were identified^15^, ^16^, ^17^, ^18^, ^19^, ^20^ that were excluded and used to screen the references for citations not captured through the search strategy; one study was identified through this process.^21^ The search was rerun in February 2022, identifying an additional 188 abstracts, with five studies meeting our inclusion criteria. This resulted in a total of 33 studies for inclusion in the review. The Cohen's κ at the title and abstract screening stage was 0.74, whereas at the full‐text review stage it was 0.86.

### 2.1 Meta‐Analysis of Studies from 2011 to 2022

#### 2.1.1 Incidence

A total of 18 studies examined the incidence of HD, with geographic representation across 12 countries (Canada,^12^ Cyprus,^10^ England,^9^, ^22^, ^23^ Iceland,^24^ Israel,^25^ Italy,^26^, ^27^, ^28^ Germany,^29^ Greece,^30^ South Korea,^31^ Spain,^32^, ^33^ Sweden,^34^ and the United States^35^, ^36^) (see Table S1 for study details).

A total of 13 studies were suitable for meta‐analysis (two from North America, one from Asia, and 10 from Europe). Five studies were excluded from the meta‐analysis as they reported incidence in select populations^22^, ^34^, ^36^ or did not provide adequate data for the calculation of cases and sample size.^10^, ^25^ The pooled incidence calculated from these studies was 0.48 cases per 100,000 person‐years (95% CI, 0.33–0.63; *I* ^2^ = 65%; *Q* = 34). Subgroup analysis by continent demonstrated a pooled incidence of 0.38 per 100,000 person‐years (95% CI, 0.27–0.49) in Europe and 1.21 per 100,000 person‐years (95% CI, 0.14–2.29) in North America, which are both significantly higher than the incidence of HD in the only study performed in Asia (see Table 1).

**TABLE 1:** Incidence of HD, 2011 to 2022

Location	Study	Cases	Sample (in person‐years)	Incidence per 100,000 person‐years	95% CI
Asia (South Korea)	Kim et al, 2015^31^	29	51,141,463	0.06	0.04–0.08
Europe (Italy)	Carrassi et al, 2017^26^	2	2,000,000	0.10	0.03–0.24
Europe (Italy)	Kodra et al, 2019^27^	614	180,588,235	0.34	0.31–0.37
Europe (Italy)	Muroni et al, 2021^28^	53	18,150,685	0.29	0.21–0.37
Europe (Germany)	Ohlmeier et al, 2019^29^	60	6,651,276	0.90	0.67–1.13
Europe (Greece)	Panas et al, 2011^30^	20	9,025,974	0.22	0.12–0.32
Europe (United Kingdom)	Sackley et al, 2011^23^	13	2,964,386	0.44	0.20–0.68
Europe (Spain)	Sienes Bailo et al, 2020^32^	4	593,387	0.67	0.01–1.34
Europe (Iceland)	Sveinsson et al, 2012^24^	8	5,714,286	0.14	0.04–0.24
Europe (United Kingdom)	Wexler et al, 2016^9^	199	29,522,583	0.67	0.58–0.77
Europe (Spain)	Vicente et al, 2021^33^	63	15,750,000	0.40	0.30–0.50
**Subgroup analysis, Europe (*I*** ^**2**^ **= 49, *Q* = 18)**				**0.38**	**0.27–0.49**
North America (United States)	Bruzelius et al, 2019^35^	267	15,198,207	1.76	1.55–1.97
North America (Canada)	Shaw et al, 2022^12^	21	3,183,874	0.66	0.38–0.94
**Subgroup analysis, North America (*I*** ^***2***^ **= 0, *Q* = 1)**				**1.21**	**0.14–2.29**
**Pooled estimate (*I*** ^***2***^ **= 65, *Q* = 34)**				**0.48**	**0.33–0.63**

Abbreviations: HD, Huntington's disease; CI, confidence interval.

### 2.2 Sensitivity Analysis

Removal of the five incidence studies that did not use valid clinical criteria or genetic testing to assess for the presence or absence of disease (ie, relied exclusively on health administrative data) resulted in a pooled incidence estimate of 0.26 per 100,000 person‐years (95% CI, 0.13–0.38; *I* ^2^ = 18%; *Q* = 8).

#### 2.2.1 Prevalence

A total of 27 studies explored the prevalence of HD, representing 21 countries (Brazil,^37^, ^38^ Cameroon,^39^ Canada,^7^, ^12^ Cyprus,^10^ Denmark,^40^ England,^5^, ^22^ Finland,^41^ Iceland,^24^ Iran,^21^ Israel,^25^ Italy,^8^, ^26^, ^42^ Germany,^29^ Greece,^30^ Northern Ireland,^43^ Scotland,^11^ South Africa,^6^ South Korea,^31^ Spain,^33^ Sultanate of Oman,^44^ Sweden,^45^ and the United States^35^, ^36^) (see Table S2 for study details).

A meta‐analysis was performed for 23 studies, of which one study^45^ provided separate prevalence rates for urban and rural jurisdictions in Sweden. Therefore, both estimates were included. Four studies were not incorporated in the meta‐analysis. Two studies did not provide adequate data to determine the number of cases detected or the total population examined,^10^, ^21^ and two studies reported the prevalence of HD in select populations.^22^, ^36^ The overall pooled prevalence of HD was 4.88 per 100,000 (95% CI, 3.38–7.06; *I* ^2^ = 49; *Q* = 47). Subanalyses by continent (Africa, Asia, Europe, North America, and South America) demonstrated that the prevalence of HD was significantly higher in Europe (6.37 per 100,000; 95% CI, 4.50–8.91) and North America (8.87 per 100,000; 95% CI, 4.69–16.78) than in Africa (0.25 per 100,000; 95% CI, 0.02–2.61) (Table 2). The subanalysis of prevalence in Asian countries included two countries from the Middle East (Israel and Oman) and one country from East Asia (Korea). If Asia is subsequently divided into the Middle East and East Asia, the prevalence of HD was significantly higher in Europe and North America than in East Asia (0.41 per 100,000; 95% CI, 0.36–0.47).

**TABLE 2:** Prevalence of HD 2011 to 2022

Location	Study	Cases	Sample	Prevalence per 100,000	95% CI
Africa (South Africa)	Baine et al, 2016^6^	384	51,489,107	0.75	0.67–0.82
Africa (Cameroon)	Cubo et al, 2017^39^	2	3,000,000	0.07	0.02–0.27
**Subgroup analysis, Africa (*Q* = 1, *I*** ^**2**^ **= 0)**				**0.25**	**0.02–2.61**
Asia (Israel)	Gavrielov‐Yusim et al, 2021^25^	69	1,580,816	4.36	3.45–5.53
Asia (South Korea)	Kim et al, 2015^31^	208	51,141,463	0.41	0.36–0.47
Asia (Sultanate of Oman)	Squitieri et al, 2020^44^	41	556,731	7.36	5.42–10.00
**Subgroup analysis, Asia (*Q* = 2, *I*** ^**2**^ **= 0)**				**2.39**	**0.33–16.56**
Europe (Italy)	Carrassi et al, 2017^26^	15	354,673	4.23	2.55–7.02
Europe (United Kingdom)	Evans et al, 2013^5^	432	3,515,986	12.29	11.18–13.50
Europe (Denmark)	Gilling et al, 2017^40^	329	5,660,000	5.81	5.22–6.48
Europe (Scotland)	Kounidas et al, 2021^11^	134	893,440	15.00	12.66–17.77
Europe (Northern Ireland)	Morrison et al, 2011^43^	180	1,698,113	10.60	9.16–12.27
Europe (Italy)	Muroni et al, 2020^42^	47	785,785	5.98	4.49–7.96
Europe (Germany)	Ohlmeier et al, 2019^29^	308	3,325,638	9.26	8.28–10.36
Europe (Greece)	Panas et al, 2011^30^	278	10,964,020	2.54	2.25–2.85
Europe (Sweden)	Roos et al, 2017 (Jamatland)^45^	28	126,765	22.09	15.25–31.99
Europe (Sweden)	Roos et al, 2017 (Uppsala)^45^	17	348,942	4.87	3.03–7.84
Europe (United Kingdom)	Sackley et al, 2011^23^	177	2,964,386	5.97	5.15–6.92
Europe (Finland)	Sipila et al, 2015^41^	114	5,377,358	2.12	1.76–2.55
Europe (Italy)	Squitieri et al, 2016^8^	34	313,341	10.85	7.75–15.19
Europe (Iceland)	Sveinsson et al, 2012^24^	3	311,114	0.96	0.31–2.99
Europe (Spain)	Vicente et al, 2021^33^	32	647,554	4.94	3.49–6.99
**Subgroup analysis, Europe (*Q* = 18, *I*** ^**2**^ **= 21)**				**6.37**	**4.50–8.91**
North America (United States)	Bruzelius et al, 2019^35^	3707	67,582,529	5.49	5.31–5.66
North America (Canada)	Fisher and Hayden, 2014^7^	631	4,609,659	13.69	12.66–14.80
North America (Canada)	Shaw et al, 2022^12^	297	3,183,874	9.33	8.33–10.45
**Subgroup analysis, North America (*Q* = 1, *I*** ^**2**^ **= 0)**				**8.87**	**4.69–16.78**
South America (Brazil)	Agostinho et al, 2015^37^	13	18,087	71.87	41.74–123.74
South America (Brazil)	Castilhos et al, 2019^38^	209	11,297,297	1.85	1.62–2.12
**Subgroup analysis, South America (*Q* = 1, *I*** ^**2**^ **= 0)**				**11.42**	**0.32–410.99**
**Total (*Q* = 47, *I*** ^**2**^ **= 49)**				**4.88**	**3.38–7.06**

Abbreviations: HD, Huntington's disease; CI, confidence interval.

### 2.3 Sensitivity Analysis

Removal of the five prevalence studies that did not use valid clinical criteria or genetic testing to assess for the presence or absence of disease (ie, relied exclusively on health administrative data) resulted in a pooled prevalence of 5.15 per 100,000 (95% CI, 3.33–7.98; *I* ^2^ = 31; *Q* = 28).

### 2.4 Risk of Bias

The mean score on the risk‐of‐bias rating was 3.5/4. Most often, studies missed points for not using valid clinical criteria or genetic testing to assess for the presence of disease (ie, relying exclusively on service codes to identify cases) or did not provide detailed estimates by subgroup or with CIs (see Table S3).

### 2.5 Combined Meta‐Analysis of Studies from 1985 to 2022

The studies identified for this review were combined with studies from the meta‐analysis in the previously published 2012 review.^4^ The overall incidence for the combined meta‐analysis was 0.47 cases per 100,000 person‐years (95% CI, 0.36–0.59; *I* ^2^ = 63%; *Q* = 44) (see Table S4). Subgroup analysis by continent revealed a significantly higher incidence of HD in Europe (0.38 per 100,000 person‐years; 95% CI, 0.28–0.49), North America (1.04 per 100,000 person‐years; 95% CI, 0.28–1.80), and Oceania (0.65 per 100,000 person‐years; 95% CI, 0.44–0.85) compared with Asia (0.08 per 100,000 person‐years; 95% CI, 0.03–0.12).

The pooled prevalence incorporating all studies from 1985 to 2022 was 3.92 cases per 100,000 (95% CI, 2.90–5.30; *I* ^2^ = 50%; *Q* = 71) (see Table S5). Subgroup analysis by continent revealed a significantly higher prevalence of HD in Europe (5.65 per 100,000; 95% CI, 4.31–7.41), North America (7.43 per 100,000; 95% CI, 4.08–13.53), and Oceania (8.61 per 100,000; 95% CI, 4.55–16.31) than in Africa (0.25 per 100,000; 95% CI, 0.02–2.61) and Asia (0.99 per 100,000; 95% CI, 0.33–2.95).

Side‐by‐side comparisons on prevalence and incidence by time period—1985 to 2010, 2011 to 2022, and 1985 to 2022—are provided in Table 3.

**TABLE 3:** Comparison of overall incidence and prevalence of HD by time period studied

Time period studied	Estimate	95% CI
Incidence		
1985–2010	0.38 per 100,000 per year	0.16–0.94
2011–2022	0.48 per 100,000 per year	0.33–0.63
1985–2022	0.47 per 100,000 per year	0.36–0.59
Prevalence		
1985–2010	2.71 per 100,000	1.55–4.72
2011–2022	4.88 per 100,000	3.38–7.06
1985–2022	3.92 per 100,000	2.90–5.30

Abbreviations: HD, Huntington's disease; CI, confidence interval.

## 3 Discussion

Since our original publication in 2012, there have been many additional studies describing the epidemiology of HD in countries around the world. The increased number of studies included and the larger sample size for the meta‐analyses performed in this study have resulted in more precise estimates of both prevalence and incidence, demonstrated by narrower CIs for the meta‐analyses combining all studies from 1985 to 2022. Comparing the point estimates from the meta‐analysis of studies from 1985 to 2010 to the meta‐analysis of studies from 2011 to 2022, the prevalence more so than the incidence of HD appears to be increasing modestly with time; however, the 95% CIs for the period estimates overlap.

Increases in the prevalence and incidence of HD may be related to an increase in the availability of molecular testing and increased recognition by physicians and patients of the HD phenotype and its variable symptom presentation. In addition, the increase in HD prevalence with time may be attributed to earlier diagnosis, advances in supportive care, and advances in the treatment of medical comorbidities, allowing affected patients to live longer. Furthermore, de novo mutations are estimated to account for 7.1% of new cases^13^ and may contribute to rising incidences.

The larger data set for these meta‐analyses allowed us to perform more subanalyses based on study continent than in our previous review. Looking at the meta‐analyses of all studies from 1985 to 2022, there is a significantly higher incidence of HD in Europe, North America, and Oceania compared with Asia and a significantly higher prevalence of HD in Europe, North America, and Oceania compared with Asia and Africa. In contrast, in our 2012 analysis, we only compared the prevalence of HD in Asia to the prevalence in Europe, North America, and Oceania combined as we did not have enough studies to perform more detailed subanalyses.

Estimates of prevalence and incidence were consistent by region, with only a few notable outliers. A study of prevalence and incidence in the United States by Bruzelius and colleagues^35^ found an incidence of HD that was much higher than other studies performed in North America and Europe, whereas the prevalence of HD found in this study was in the expected range, suggesting a potential problem with the procedure used to identify incident cases. One study of the prevalence of HD in South America^37^ found a prevalence five to 10 times higher than all other studies in South America, North America, and Europe; this rural region studied in Brazil is known to have a geographical cluster of people affected by HD. Subanalysis by continent was problematic for Asia, as the prevalence and incidence of HD in countries situated in the Middle East were noticeably different than countries in East Asia. The Middle East prevalence and incidence estimates were more like estimates in Europe, North America, and Oceania, whereas the estimates in East Asia were much lower.

Comparatively less is known about HD in Africa. Squitieri and colleagues analyzed a large Omani family HD cluster and identified a unique C6xC9 haplotype that was traced back to an ancestor of sub‐Saharan African origin, in contrast to three unrelated Omani parent and offspring HD trios that had HD allele expansions on haplotype A, similar to European populations.^46^ A study genotyping HD in South Africa found distinct HD allele haplotypes: Caucasian and mixed patients were mostly associated with haplogroup A, similar to European populations, whereas in Black patients, there were associations with haplogroups B and C, implying a distinct genetic origin.^47^ Although further work is needed to understand HD epidemiology in Africa and the Middle East, there seem to be distinct genetic origins, and the work on the Omani cluster may suggest a spread of HD from Africa to the Middle East.

Our post hoc sensitivity analysis that eliminated studies that relied strictly on health administrative data without the use of valid clinical criteria or genetic testing to confirm the presence of disease suggested that the inclusion of such studies may overestimate the incidence of HD, whereas there was little effect on the prevalence point estimate. HD is not the only condition for which this trend has been observed; a systematic review of incidence studies of Guillain‐Barré syndrome also found that studies using administrative data without clinical confirmation of disease status reported higher incidence estimates.^48^ These higher incidence estimates may be the result of inaccuracies in coding or diagnosis. Why such inaccuracies should affect incidence estimates specifically is uncertain.

Like our previous review, all but one study identified individuals with HD through a positive genetic test result, medical records, disease registries, or administrative databases. Door‐to‐door population‐based studies of this condition are not feasible because of the large sample size required to make precise estimates given the low prevalence of the condition. There is very little information on the validity of administrative data for identifying people with HD and the accuracy of case definitions. Two of the studies included in this review assessed and compared the incidence of HD using multiple data sources. Kodra and colleagues used the Italian National Rare Diseases Registry (NRDR) and the National Hospital Discharge Database (NHDD).^27^ Using the NRDR as an example, the study examined the proportion of HD cases identified in the NRDR that were also identified in the NHDD as an estimate of the NHDD completeness and found a sensitivity of 0.52 (95% CI, 0.49–0.55). The accuracy of the NHDD, assessed by determining the positive predictive value (the proportion of cases identified in the NHDD that were also present in the NRDR) was 0.48 (95% CI, 0.45–0.52). Using the population in Navarre, Spain, Vicente and colleagues sought to validate the accuracy and sensitivity of HD diagnostic codes in routinely collected health care information system data sets (Minimum Basic Data Set at Hospital Discharge, Electronic Clinical Records in Primary Care, Temporary Work Disability Registry, and Mortality Statistics) compared with medical records as the gold standard.^33^ The combined health information system data sets yielded a positive predictive value of 71.8% (95% CI, 59.7–81.6) and a sensitivity of 82.2% (95% CI, 70.1–90.4), with substantial variability in sensitivity among individual data sets. As a result of these findings, there is a definite need for case validation studies in HD studies using health administrative data to ensure that estimates of prevalence and incidence are not based on misclassified cases. The lack of validation data is a major limitation of all included studies and the results of this systematic review.

Using a different approach to estimate the diagnosed prevalence of manifest HD, Crowell and colleagues used published incidence data from eight countries and natural history data from the Enroll‐HD observational study to model the prevalence of HD by disease stage.^49^ Diagnosed prevalence was estimated to be 8.2 to 9.0 per 100,000 in the United States, Canada, and five European countries (France, Germany, Italy, Spain, and the United Kingdom), in which 63% of cases were in Shoulson‐Fahn stages 1 and 2. These estimates are generally concordant with published estimates of prevalence but depend on the accuracy of the incident data they are derived from and the assumption that the estimated stage at incidence, time in stage, and time at death are constant.

Overall, the present study suggests that there may be a small increase in both the global prevalence and incidence of HD since the previously performed systematic review and meta‐analysis in 2012,^4^ but this increase is not statistically significant. Although a similar proportion of the studies included for meta‐analysis used molecular testing as part of their case ascertainment criteria in the current review compared with 2012, the updated meta‐analysis naturally includes additional and more recent studies incorporating genetic testing. Future studies should use multisource ascertainment of HD cases to capture cases of HD and explore its epidemiology with precision and accuracy. As HD is not a common disease, many individuals with HD receive care at regional centers of expertise, with diagnostic molecular testing performed at a single institution. Multisource ascertainment would permit further case validation studies to be performed and determine the sensitivity and specificity of diagnostic codes used in health administrative data to capture HD cases.

## Author Roles

1. Research Project: A. Conception, B. Organization, C. Execution; 2. Manuscript: A. Writing of the First Draft, B. Review and Critique.

A.M.: 1C, 2A, 2B

Y.M.: 1C, 2A, 2B

L.S.: 1B, 2B

T.P.: 1B, 1C, 2A, 2B

## Financial Disclosures

L.S. is employed by the Public Health Agency of Canada. T.P. has received research funding from Alberta Health, the Alberta Children's Hospital Research Institute, and the Public Health Agency of Canada. A.M. and Y.M. have no disclosures.

## Supporting information


**Appendix S1.** Supporting information

## Data Availability

The data that supports the findings of this study are available in the supplementary material of this article.
