# Supplementary material for: Prevalence and Incidence of Huntington's Disease: An Updated Systematic Review and Meta‐Analysis
Source: Mov Disord. 2022 Sep 26;37(12):2327–35. doi: 10.1002/mds.29228 (PMC10086981; doi:10.1002/mds.29228)
Supplement: Supplementary file 1 — Appendix S1. Supporting information [file MDS-37-2327-s001.docx]

**Appendix 1A. Complete search strategy in OVID Medline.**

1. exp Huntington Disease/cl, di, ep, et [Classification, Diagnosis, Epidemiology, Etiology]

2. Huntington*.tw,kf.

3. 1 or 2

4. exp Incidence/

5. Incidence*.tw,kf.

6. 4 or 5

7. exp Prevalence/

8. prevalence*.tw,kf.

9. 7 or 8

10. exp Epidemiology/cl, di, mt, sn [Classification, Diagnosis, Methods, Statistics & Numerical Data]

11. epidemiology*.tw,kf.

12. 10 or 11

13. 6 or 9 or 12

14. 3 and 13

15. *Huntington Disease/ep [Epidemiology]

16. 14 or 15

17. limit 16 to yr="2010 -Current"

18. limit 17 to animals

19. 17 not 18

**Appendix 1B. Complete search strategy in EMBASE.**

1. exp Huntington chorea/di, ep, et [Diagnosis, Epidemiology, Etiology]

2. Huntington*.tw,kw.

3. exp incidence/ep [Epidemiology]

4. Incidence*.tw,kw.

5. exp prevalence/ep [Epidemiology]

6. prevalence*.tw,kw.

7. exp epidemiology/

8. epidemiology*.tw,kw.

9. 3 or 4 or 5 or 6 or 7 or 8

10. 1 or 2

11. 9 and 10

12. Huntington chorea/ep

13. 11 or 12

14. limit 13 to yr="2010 -Current"

15. limit 14 to animals

16. 14 not 15

**Supplementary Table 1. Incidence Studies**

| Study, Date, Country | Population | Cases | Data Source | Diagnosis established by | Incidence Date | Overall Calculated Incidence Rate | Subgroups Calculated Incidence Rate |
| --- | --- | --- | --- | --- | --- | --- | --- |
| Bruzelius, 2019, U.S. | All ages  Total Person Years  2004  n=12,926,069  2005 n=13,516,341  2006 n=13,921,818  2007 n=14,011,451  2008 n=13,517,434  2009 n=13,066,931  2010 n=12,666,205  2011 n=12,811,864  2012 n=13,015,802  2013 n=13,276,662  2014 n=12,721,108  2015 n=13,564,544  2016 n=15,198,207  Total n=174,214,435 | 2004=117  2005=136  2006=197  2007=184  2008=203  2009=234  2010=181  2011=243  2012=231  2013=245  2014=245  2015=294  2016=267  Total=2777 | Administrative claims data base of privately insured enrollees in the United States (Optum Clinformatics Datamart) | Identified patients diagnosed with HD according to ICD-9 or ICD-10 codes. | 2004-2016 | Crude cumulative incidence rate: 1.59/100,000/person years (95% CI 1.53-1.65)  Age standardized cumulative incidence rate: 1.22/100,000/person years (95% CI 1.06-1.15)  Annual incidence per 100,000 Person Years, 95% CI  2004 0.91, 0.44-1.07  2005 1.01, 0.84-1.18  2006 1.42, 1.22-1.61  2007 1.31, 1.12-1.50  2008 1.50, 1.30-1.71  2009 1.79, 1.56-2.02  2010 1.43, 1.22-1.64  2011 1.92, 1.66-2.14  2012 1.77, 1.55-2.00  2013 1.85, 1.61-2.08  2014 1.93, 1.68-2.17  2015 2.17, 1.92-2.42  2016 1.76, 1.55-2.42  Age adjusted annual incidence per 100,000 Person Years, 95% CI  2004 0.97, 0.81-1.13  2005 0.66, 0.55-0.77  2006 0.86, 0.74-0.98  2007 0.79, 0.68-0.90  2008 1.13, 0.97-1.29  2009 1.23, 1.07-1.39  2010, 0.87, 0.74-1.00  2011 1.15, 1.00-1.30  2012 1.24, 1.08-1.40  2013 1.26, 1.10-1.42  2014 1.31, 1.15-1.47  2015 1.68, 1.49-1.87  2016 1.18, 1.04-1.32 |  |
| Carassi, 2017, Italy | All ages  <25= 68,713  25–34=49,734  35–44=52,539  45–54=50,031  55–64=49,587  65–74=45,224  75+=38,168  Total=353,996  Men=169,334  Women= 184,662 | <25= 0  25–34=2  35–44=5  45–54=7  55–64=6  65–74=0  75+=2  Total=22  By year  1990-94 =5  1995-99=8  2000-04=7  2005-09=2 | Chart review from archives and outpatient records from the Units  of Neurology, Ferrara University Hospital, anonymous data supplied  by the Medical Genetics Section and from administrative  data from the Hospital Health Statistics Office.  Regional Record for Rare Diseases, using disease identification  ICD-9M code 333.4 and the medical fare exemption code RF0080. | Any individual carrying CAG expansion  on the HTT gene and presenting onset with the unequivocal  presence of an otherwise unexplained extrapyramidal movement  disorder. | 1990–2009 | 0.3 per 100,000 person years (95% CI 0.2–0.5) | By age  <25= -  25–34=0.2 per 100,000 person years  35–44=0.5 per 100,000 person years  45–54=0.7 per 100,000 person years  55–64=0.6 per 100,000 person years  65–74=-  75+=0.3 per 100,000 person years  By gender  Female= 0.3 per 100,000 person years (95%CI0.1–0.5)  Male= 0.3 per 100,000 person years (95%CI0.1–0.5)  By quinquennia  1990–1994= 0.3 per 100,000 person years (95%CI 0.09–0.7)  1995–1999 = 0.5 per 100,000 person years (95%CI 0.19–0.89)  2000–2004= 0.4 per 100,000 person years (95%CI 0.16–0.82)  2005–2009= 0.1 per 100,000 person years (95%CI 0.01–0.04) |
| Demetriou, 2018, Cyprus | All ages  Total population of Cyprus in 2015 according to population census data from Cyprus Statistical Service (number not provided) | 58 cases of HD identified | Neurogenetic Data Bank of the Bi-communal Cyprus institute of Neurology and Genetics | Clinical diagnosis of HD by neurologist and molecular diagnosis of HD | 2015 | 0.12 per 100,000 per year (95%CI 0.00-0.66)  Crude average annual incidence from 1984-2015 0.25 per 100,000 per year (95%CI 0.19-0.33) |  |
| Douglas, 2013, UK | Juvenile HD, defined as those under the age of 21 at disease onset  Population divided by groups  0–4=4,097,551  5–9=4,156,414  10–14=4,115,431  15–20=4,762,455 | 12 cases total identified  0-4; 0 cases  5-9; 3 cases  10-14; 3 cases  15-20; 6 cases | General Practice Research Database | UK read codes for Huntington’s chorea and dementia in Huntington’s disease | 1990-2010 | 0.70 (95% CI 0.36-1.22) per 1,000,000 | Age (per million)  0–4=0 (0 to 1.1)  5–9=0.7 (0.2 to 2.1)  10–14=0.7 (0.2 to 2.1)  15–20=1.3 (0.5 to 2.7) |
| Gavrielov-Yusim, 2021, Israel | Patients receiving care from Maccabi Health Services (MHS) from 2000 to 2019, an insurer and healthcare provider for a quarter of Israel’s population  All adult patients (18 years and older)  1,580,816 MHS members in 2018 | Annual new HD case counts fluctuated from 2 to 13 between 2000 and 2018. Number of case per year and population not provided in paper. | Database of MHS. MHS collects diagnoses from hospitals, general practice, specialty clinics, ambulatory clinics, and hospice settings. MHS captures data on medical procedures, imaging, and drug treatments through drug purchases and pharmacy dispensations. All data are linked at the patient level using a unique national patient identifier. | Data were extracted for all adult MHS patients with an HD diagnosis defined by at least one record of ICD-9 code 333.4. All diagnoses included in the study were made by a neurologist or were documented as a chronic disease diagnosis in the patient’s medical record.  Patients diagnosed with drug induced chorea, subacute dyskinesia due to drugs, Sydenham’s chorea or rheumatic chorea after the index HD diagnosis were excluded. | Annual incidence defined as the number of new adult HD cases per year between 2000 and 2018. | Annual incidence fluctuated from 0.17 to 1.34 per 100,000 from 2000 to 2018. Incidence by year not reported. | No further data reported on incidence |
| Gordon, 2016, US | Navajo residents of the Navajo nation  N=275,000 | 0 | The Indian  Health Service National Patient Information Reporting  System | Unspecified | 2001–2011 | 0 |  |
| Kim, 2015, South Korea | All ages  2009 n=49,773,145  2010 n=50,515,666  2011 n=50,734,284  2012 n=50,948,272  2013 n=51,141,463 | 2009=99 (year registry started)  2010=36  2011=19  2012=25  2013=29 | Databases of the Rare Diseases Registry and National Health Insurance | For Rare Disease Registry – patients with physician-certified diagnosis  For National Health Insurance - unspecified | 2009-2013 | Mean of calculated crude annual incidence rates was 0.06 per 100,000 persons per year  2009=(year registry was started)  2010=0.07/100,000  2011=0.04/100,000  2012=0.05/100,000  2013=0.06/100,000 |  |
| Kodra, 2019, Italy | All ages  n=not reported | 614 cases of HD recorded in National Rare Disease Registry from 2012-2014 | Italian National Rare Diseases Registry  Hospital Discharge Database of the Italian Ministry of Health | For National Rare Disease Registry – unclear  For Hospital Discharge Database – use of ICD9 codes for HD in diagnostic fields of database | 2012-2014 | National Rare Diseases Registry  =0.34 per 100,000 per year (no specific year data or CI provided)  Hospital Discharge Database=0.37 per 100,000 per year | National Rare Diseases Registry  Crude rate (per 100,000 per year)  0–10 years= 0.02  11-20= 0.01  21-30= 0.10  31-40=0.28  41-50=0.58  51-60=0.67  61-70=0.55  71-80=0.42  >90=0.07  Total = 0.34  Hospital Discharge Database  Crude rate (per 100,000 per year)  0–10 years= 0.05  11-20= 0.06  21-30= 0.10  31-40=0.27  41-50=0.44  51-60=0.52  61-70=0.66  71-80=0.73  >90= 0.45  Total 0.37 |
| Muroni, 2021, Sardinia, Italy | All ages  Population on January 1, 2009: 1,672,404  Population on December 31, 2018: 1,639,591  Average 1,663,859 at the middle of the period | 53 individuals residing in Sardinia diagnosed with clinically manifest HD during the incidence period | One genetic laboratory providing genetic analysis for HD and the two tertiary referral centres for HD in Sardinia. Additional cases actively sought through contact with other public neurologic institutions in Sardinia and neurologists from tertiary referral centres in Italy where Sardinian HD patients could likely have been evaluated | Incident cases defined as individuals with phenotype suggesting HD (i.e. chorea and/or psychiatric signs and/or cognitive decline) & greater than 36 CAG repeats in *HTT,* who resided in the study area at time of clinical onset between January 1, 2009 and December 31, 2018 | January 1, 2009- December 31, 2018 | Average annual incidence rate of 2.92 per 1,000,000 person-years (95% CI 2.2-3.9) | Sex :  Women : 3.9 per 1,000,000 person-years (95% CI 2.7-5.5)  Men : 1.8 per 1,000,000 person-years (95% CI 1.0-3.0)  By province :  Cagliari : 1.9 per 1,000,000 person-years (95% CI 1.1-3.9)  South Sardinia : 6.3 per 1,000,000 person-years (95% CI 4.2-9.5)  Oristano : 0.6 per 1,000,000 person-years (95% CI 0.1-3.5)  Nuoro : 3.2 per 1,000,000 person-years (95% CI 1.6-6.7)  Sassari : 2.2 per 1,000,000 (95% CI 1.2-3.9) |
| Ohlmeier, 2019, Germany | All ages  Claims data from approximately 4,000,000 insured persons from 70 German statutory health insurances  3,325,638 people | Number of incident cases not reported | Applied Health Research Berlin Research  Database | At least two outpatient or inpatient ICD-10 codes for HD during the study period were required for case identification | January 2015 to 31 December 2016 | 2 year incidence  1.8 per 100,000 persons (95%CI 1.4–2.4) |  |
| Panas, 2011, Greece | All ages  Population  n= 10,964,020 | 278 cases | Neurogenetics Unit, Athens Greece | Molecular diagnosis of HD | January 1995 to December 2008 | 2.2 per 1,000,000 per year |  |
| Sackley, 2011, UK | All ages  2004 n= 2,840,202  2005 n=2,948,206  2006 n=2,977,044  2007 n= 2,983,870  2008 n= 2,964,386 | 324 cases identified during the study period | The Health Improvement Network database | Patients were identified as diagnosed with HD if they had a Read code indicating the condition. Read codes indicating a possible diagnosis (other, unspecified chorea) and a family history of the condition were also identified. | December 31, 2004 to 2008 | 2004  0.70/100,000/yr  2005  0.78/100,000/yr  2006  0.64/100,000/yr  2007  0.50/100,000/yr  2008  0.44/100,000/yr |  |
| Shaw, 2022, Alberta, Canada | Age ≥21 years  Estimated mid-year Alberta adult (≥21 years), in person-years:  2010/11: 2,745, 527  2011/12: 2,739,009  2012/13: 2,862,175  2013/14: 2,947,228  2014/15: 3.027,608  2015/16: 3,070,416  2016/17: 3,104,520  2017/18: 3,139,143  2018/19: 3,183,874  Average 5-yr (2014/15-2018/19): 15,525,561 | 222 incident cases over the study period (2010/11 to 2018/19)  129 cases over the last 5 years (2014/15-2018/19)  21 cases in the final year (2018/19) | De-identified province-wide administrative health data released by the Government of Alberta Ministry of Health from the following linkable administrative datasets: Alberta Blue Cross Pharmacy Claims, Alberta Continuing Care Information System, Alberta Precision Laboratories Dataset, Diagnostic Imaging Dataset, Discharge Abstract Database, National Ambulatory Care Reporting System, Pharmaceutical Information Network Dispenses, Population Registry, Practitioner Claims, and Vital Statistics (deaths).  Data from Practitioner Claims, Discharge Abstract Database, and National Ambulatory Care Reporting System datasets requested from April 1, 1997 to facilitate identification of incident & prevalent cases | Cases required 2 diagnostic codes within a two-year span from ICD-9-CM or ICD-10 -CA, appearing in any position in the Discharge Abstract Database or National Ambulatory Care Reporting System.  Index dates within the case ascertainment period (April 1, 2010 to March 31, 2020) were considered incient cases. | April 1, 2010 to March 31, 2020 | Average 5-year (2014/15-2018/19): 0.83 per 100,000 person-years  (95% CI not reported)  2018/19:  0.66 per 100,000 person-years  (95% CI not reported) | Incidence by year (per 100,000 person-years)  2010/11: 0.95  2011/12: 0.47  2012/13: 0.91  2013/14: 0.95  2014/15: 0.86  2015/16: 1.21  2016/17: 0.71  2017/18: 0.73  2018/19: 0.66  95% CI not reported |
| Sienes, 2020, Spain | All ages  Aragon population 1,300,000  Population by year not reported  Total population served by Clinical Genetics Section during the full period was 593,387 person-years on average | 50 incident cases identified during study period | Clinical Genetics Section of the Hospital Universitario Miguel Servet | Patients seen in clinical genetics section who had HD diagnosis based on genetic testing | 2007–2019 | 0.648/100,000 person-years  (95% CI 0.587 to 0.709) | Females 2.23/100,000 ( 95% CI 1.63 to 2.83)  Males  1.62/100,000 (95% CI 0.86 to 2.38) |
| Sveinsson, 2021  Iceland | All ages  n=311,114  Population by year not reported | 8 individuals diagnosed with HD from 1988 to 2007 | Multiple data sources: medical records and discharge diagnoses from all hospitals in Iceland, including records from neurological, psychiatric and genetic departments. Interviews with family members and physicians. | Based on presence of hyperkinetic movement disorder, psychiatric symptoms, progressive cognitive decline, and family history of first degree relative with HD or positive DNA test for HD. | (1988–2007) | 0.14 cases per 100,000 person-years |  |
| Vicente, 2021, Navarre, Spain | 647,554 inhabitants in January 2018 | 1991-1999 = 22  2000-2008 = 21  2009-2017 = 20  Total incident cases = 63 | Multiple data sources:  Minimum Basic Data Set at Hospital Discharge (MBDS): all episodes containing the ICD-9-CM code 333.4 until 2015, and the ICD-10-ES (Spanish modification of ICD version 10) code G10 from 2016-2017  Electronic Clinical Records in Primary Care (ECRPC): “Huntington disease, chorea” descriptors  Temporary Work Disability Registry (TWDR): all episodes containing the ICD-9-CM code 333.4 from 2000-2017  Medical Genetics Centre: patients with clinical signs of HD and relatives referred for assessment and testing | Case validation using medical records and clinical assessment by a neurologist and clinical geneticist with HD expertise.  1 of the following inclusion criteria for diagnosis of HD:  1) neurocognitive signs compatible with HD and a genetic test result of >35 CAG repeats in the *HTT* gene  2) neurocognitive signs compatible with HD, without a genetic test result but with a genetically confirmed family history | 1991-2017  Incidence reported in 9-year intervals: 1991-1999; 2000-2008; 2009-2017 | Total:  0.40 per 100,000 (95% CI 0.31-0.50)  Annual incidence:  1991-1999: 0.46 per 100,000 (95% CI 0.30-0.69)  2000-2008: 0.39 per 100,000 (95% CI 0.25-0.59)  2009-2017: 0.35 pre 100,000 (95% CI 0.22-0.53) | Sex :  Men: 0.37 per 100,000 (95% CI 0.25-0.52)  Women : 0.43 per 100,000 (95% CI 0.30-0.59)  Age :  ≤20 : 0.03 per 100,000 (95% CI 0-0.16)  21-40 : 0.19 per 100,000 (95% CI 0.10-0.35)  41-60 : 0.63 per 100,000 (95% CI 0.42-0.91)  >60 : 0.78 per 100,000 (95% CI 0.52-1.12) |
| Wandell, 2021, Sweden | All ages  First generation immigrants n=6,042,891  Second generation immigrants n=4,860,469  Number of person-years not provided | First generation: 1034 incident cases  Second generation: 1001 incident  cases | Swedish Total Population Register and the National Patient Register | Huntington’s disease diagnostic code from the registry. Registry includes diagnoses for hospitalized patient and out-patient diagnoses from specialist care. | January 1, 1998, until  December 31, 2015 | First generation immigrants  age-standardized incidence  Men  0.73/100,000 person-years  Women  0.81/100,000 person-years  Second generation Immigrants not reported | Swedish-born 0.82/100,000 person years  Foreign-born  0.53/100,000 person years |
| Wexler, 2016, UK | 21 years or older registered in the Clinical Practice Research Datalink  n=9,282,126 (corresponding to 54,907,468 person-years)  1990–1996  6,778,613 patient-years  1997–2003  18,533,173 patient-years  2004–2010  29,522,583 patient-years | 393 | The Clinical Practice Research Datalink. | Incident patients were defined as all those with a first  record of an HD diagnostic code (UK Read Codes), during the observation period with at least 12 months  elapsed since their registration date and t two recorded  contacts with their contributing practice prior to the HD diagnosis.  The Read codes used to identify cases  of HD were F134.00 (Huntington’s chorea) and  Eu02200 (dementia in HD). | 1990 to 2010 | 7.2/1,000,000 (95% CI 6.5 to 7.9) | Females 7.1/1,000,000 patient-years (95% CI 6.1 to 8.10)  Males 7.3/1,000,000 patient-years (95% CI 6.3 to 8.4)  5–6-year bands  1990–1996 8.26/1,000,000 patient-years (95% CI 6.24 to 10.73)  1997–2003  7.45/1,000,000 patient-years (95% CI 6.26 to 8.80)  2004–2010  6.7/1,000,000 patient-years (95% CI 5.84 to 7.75)  Incidence and age of onset  1990–1996  <40: 7.9/1,000,000 patient-years (95% CI 4.4 to 13.1)  40–49: 13.2/1,000,000 patient-years (95% CI 7.0 to 22.6)  50–59: 14.8/1,000,000 patient-years (95% CI 7.6- 25.8)  >60:  10.0/1,000,000 patient-years (95% CI 5.7 to 16.2)  1997–2003  <40:  5.6/1,000,000 patient-years (95% CI 3.7 to 8.1)  40-49:  11.8/1,000,000 patient-years (95% CI 8.0 to 16.8)  50-59:  13.1/1,000,000 patient-years (95% CI 9.0 to 18.4)  >60:  10.7/1,000,000 patient-years (95% 7.8 to 14.3)  2004–2010  <40: 6.6/1,000,000 patient-years (95% CI 4.9 to 8.7)  40-49:  10.8/1,000,000 patient-years (95% CI 8.0 to 14.3)  50-59:  7.2/1,000,000 patient-years (95% CI 4.8 to 10.4)  >60:  10.3/1,000,000 patient-years (95% CI 8.1 to 13.0) |

**Supplementary Table 2. Prevalence Studies**

| Study, Date, Country | Population | Cases | Data source | Diagnosis established by | Prevalence Date | Overall Calculated Prevalence | Subgroups Calculated Prevalence |
| --- | --- | --- | --- | --- | --- | --- | --- |
| Agostinho, 2015, Brazil | All ages  symptomatic individuals referred for genetic testing  or at genetic risk for HD  n=18,087 | 13 | Survey to individuals with a clinical condition suggestive of HD, or being part of a risk group for HD. | Genetic testing performed the day of the survey. | January 2011-June 2013 | 7.2/10,000 | Not Reported |
| Baine, 2016, South África | All ages  All genetically confirmed HD cases  n=51,000,000 | Total HD cases=384  Black=71  Mixed ancestry=79  White=234  Total HDL2 cases=52  Black= 34  Mixed ancestry=18  White=0 | Database records reviewed retrospectively over a 20 year period to identify all individuals referred for HD testing; clinical files were also reviewed where available | Genetic testing | 1995-2014 | Prevalence of HD and HDL2 reported together  Black 0.25 per 100,000  Mixed ancestry 2.10 per 100,000  White 5.10 per 100,000 | Not Reported |
| Bruzelius, 2019, US | All ages  n=67,582,529 | 3,707 | Administrative claims data base of privately insured enrollees in the United States (Optum Clinformatics Datamart) | Identified patients diagnosed with HD according to ICD-9 ICD-10 codes | 2003-2016 | Diagnostic frequency per 100,000 persons: 5.49/100,000 (95% CI 5.31-5.66)  Age adjusted diagnostic frequency per 100,000 person: 6.52/100,000 (95% CI 6.42-6.62) | Gender  Female 6.14 (95%CI 5.88-6.40)  Age adjusted 7.05 (95%CI 6.91-7.19)  Male 4.81 (95%CI 4.58-5.05)  Age adjusted 6.10 (95%CI 5.96-6.24)  Race  Asian 2.08 (95%CI 1.53-2.62)  Age adjusted 3.58 (95%CI 3.19-3.97)  Black 5.65 (95%CI 5.02-6.27)  Age adjusted 7.38 (95%CI 7.03-7.73  Hispanic 2.93 (95%CI 2.53-3.32)  Age adjusted 4.34 (95%CI 4.09-4.59)  White 6.73 (95%CI 6.47-6.99)  Age adjusted 7.76 (95%CI 7.63-7.89)  Annual income  <49,000 8.52 (95%CI 7.91-9.14)  Age adjusted 8.32 (95%CI 8.00-8.64)  50,000-99,000  7.39 (95%CI 6.88-7.89)  Age adjusted  7.59 (95%CI 7.41-7.77)  >100.000  4.79 (95% CI 4.39- 5.18)  Age adjusted 6.54 (95% CI 6.37-6.71)  Educational attainment  High school or less  5.86 (95%CI 5.51-6.22)  Age adjusted  7.08 (95%CI 6.89-7.27)  Some college  6.37 (95%CI 6.08-6.66)  Age adjusted 7.16 (95%CI 6.96-7.36)  College or professional  4.37 (95%CI 3.95-4.79)  Age adjusted 5.98 (95%CI 5.74-6.22) |
| Castilhos, 2019, Brazil, Rio Grande do Sul | All ages  Invited to participate symptomatic patients with HD and those at genetic risk of developing HD.  Population of Rio Grande do Sul not provided. | 209 symptomatic HD patients, 690 individuals at 50% risk, 515 individuals at 25% risk | Database and medical charts | Genetic testing | September 2013-December 2016 | 1.85/100,000 | Not Reported |
| Carrassi, 2017, Italy | All ages  Total=354,673  Men =169,510 Women=185,163  <25= 71,754  25–34=40,208  35–44=61,956  45–54=59,991  55–64=52,550  65–74=42,153  75+=26,061 | Total=15  Male=8  Female=7 | Chart review from archives and outpatient records from the Units  of Neurology, Ferrara University Hospital, anonymous data supplied  by the Medical Genetics Section and from administrative  data from the Hospital Health Statistics Office.  Regional Record for Rare Diseases, using disease identification  ICD-9M code 333.4 and the medical fare exemption code RF0080. | Any individual carrying CAG expansion  on the HTT gene and presenting onset with the unequivocal  presence of an otherwise unexplained extrapyramidal movement  disorder. | December 31, 2014 | 4.2/100,000 (95% CI 2.0-7.0) | By age  <25= -  25–34= 2.5 per 100,000 persons  35–44=-  45–54=6.7 per 100,000 persons  55–64=5.7 per 100,000 persons  65–74=11.9 per 100,000 persons  75+=7.7 per 100,000 persons  By gender  Female= 4.7 per 100,000 person (95%CI 2-9)  Male= 3.8 per 100,000 person (95%CI 2-7) |
| Cubo, 2017, Cameroon | All ages  Inpatient/outpatient setting covered an urban population of 3,000,000 people | 2 | Patients with movement disorders were identified from a registry of neurological disorders in outpatient and inpatient settings from two urban public hospitals in Douala and two rural health care centers in Cameroon | Chart review; neurological diagnoses coded according to ICD-10, Huntington’s disease G10 | May 2012-May 2014 | 2 cases of HD identified from population of 3,000,000 | Not Reported |
| Dastgiri, 2012, Iran | All ages  n=not reported | NR | Molecular diagnosis | identified in the  Departments of Molecular Genetics and Epidemiology  of Tabriz University of Medical Sciences, Tabriz, Iran. | January 2005 and December 2009 | 0.196/100,000 (95% CI 0.05-0.34) | Not Reported |
| Demetriou, 2018, Cyprus | All ages  Total population of Cyprus in 2015 according to population census data from Cyprus Statistical Service (number not provided) | 58 cases of HD identified | Neurogenetic Data Bank of the Bi-communal Cyprus institute of Neurology and Genetics | Clinical diagnosis of HD by neurologist and molecular diagnosis of HD | 2015 | 4.64 per 100,000  95% CI 3.30-6.34 | Not Reported |
| Douglas, 2013, UK | Juvenile HD, defined as those under the age of 21 at disease onset  1990 n=248518  1991 n=304836  1992 n=350401  1993 n=376180  1994 n=406351  1995 n=434286  1996 n=524798  1997 n=605201  1998 n=708142  1999 n=850823  2000 n=946889  2001 n=1016667  2002 n=1075286  2003 n=1104342  2004 n=1133156  2005 n=1153294  2006 n=1176419  2007 n=1188555  2008 n=1184231  2009 n=1175793  2010 n=1167683 | 21  1990=1  1991=1  1992=1  1993=5  1994=5  1995=6  1996=6  1997=6  1998=6  1999=7  2000=6  2001=6  2002=7  2003=8  2004=10  2005=8  2006=6  2007=7  2008=8  2009=3  2010=3 | General Practice Research Database | UK read codes for Huntington’s chorea and dementia in Huntington’s disease | 1990-2010 | 6.77/1,000,000 (95%CI 5.6-8.12)  Prevalence by year  1990=4.0 (0.1 to 22.4)  1991=3.28 (0.1 to 18.3)  1992=2.9 (0.1 to 15.9)  1993=13.3 (4.3 to 31.0)  1994=12.3 (4.0 to 28.7)  1995=13.8 (5.1 to 30.1)  1996=11.4 (4.2 to 24.9)  1997=9.9 (3.6 to 21.6)  1998=8.5 (3.1 to 18.4)  1999=8.2 (3.3 to 17.0)  2000=6.3 (2.3 to 13.8)  2001=5.9 (2.2 to 12.9)  2002=6.5 (2.6 to 13.4)  2003=7.2 (3.1 to 14.3)  2004=8.8 (4.2 to 16.2)  2005=6.9 (3.0 to 13.7)  2006=5.1 (1.9 to 11.1)  2007=5.9 (2.4 to 12.1)  2008=6.8 (2.9 to 13.3)  2009=2.6 (0.5 to 7.5)  2010=2.6 (0.5 to 7.5) |  |
| Evans, 2013, UK | All patients aged 21 years or more who registered with general practices contributing to the GPRD  1990 n=663,493  1991 n=899,206  1992 n=1,012,540  1993 n=1,155,285  1994 n=1,250,401  1995 n=1,334,767  1996 n=1,507,407  1997 n=1,800,025  1998 n=2,003,819  1999 n=2,358,898  2000 n=2,812,169  2001 n=3,065,401  2002 n=3,308,580  2003 n=3,397,539  2004 n=3,510,661  2005 n=3,599,761  2006 n=3,603,973  2007 n=3,628,381  2008 n=3,610,472  2009 n=3,591,467  2010 n=3,515,986 | 1136 | UK’s General Practice Research Database (GPRD) | Eligible cases defined as persons with one or more recorded diagnoses of HD or Huntington’s chorea anywhere in their medical records.  UK read codes to identify the cases were for Huntington’s chorea and dementia in Huntington’s disease | 1990-2010 | Average prevalence of HD between 1990 and 2010: 10.0/100,000 (95%CI 8.8-11.3)  1990 5.4/100,000 (95%CI 3.8-7.5)  1991 6.3/100,000 (95%CI 4.8-8.2)  1992 6.4/100,000 (95%CI 4.6-8.2)  1993 6.4/100,000 (95%CI 5.0-8.0)  1994 7.5/100,000 (95%CI 6.1-9.2)  1995 7.2/100,000 (95%CI 5.8-8.8)  1996 7.1/100,000 (95%CI 5.8-8.6)  1997 7.9/100,000 (95%CI 6.7-9.4)  1998 8.1/100,000 (95%CI 6.9-9.4)  1999 8.4/100,000 (95%CI 7.3-9.7)  2000 8.5/100,000 (95%CI 7.4-9.6)  2001 9.3/100,000 (95%CI 8.3-10.4)  2002 9.6/100,000 (95%CI 8.6-10.7)  2003 9.9/100,000 (95%CI 8.8-11)  2004 11.0/100,000 (95%CI 9.9-12.2)  2005 11.3/100,000 (95%CI 10.2-12.4)  2006 11.4/100,000 (95%CI 10.4-12.6)  2007 11.3/100,000 (95%CI 10.2-12.4)  2008 12.1/100,000 (95%CI 11.1-13.4)  2009 12.4/100,000 (95%CI 11.3-13.6)  2010 12.3/100,000 (95%CI 11.2-13.5) | By 7 years period  (values per 100,000)  1990-1996  21-39= 5.1(95%CI 3.8-6.7)  40-49=12(95%CI 9.1-15.5)  50-59=16.5 (95%CI 17.8-21.1)  60-69=12.6 (95%CI 9.1-17.1)  >70= 7.2(95%CI 4.8-10.4)  1997-2003  21-39= 5.6(95%CI4.5-6.5)  40-49=14.1(95%CI 11.0-16.5)  50-59=18.5 (95%CI 15.9-21.4)  60-69=18.5 (95%CI 15.5-21.9)  >70 10.8(95%CI 8.7-13.3)  2004-2010  21-39= 6.0 (95%CI 5.1-7.1)  40-49=17.9(95%CI 15.8-20.3)  50-59=22.0 (95%CI 19.4-24.9)  60-69=24.2 (95%CI 21.1-27.5)  >70= 15.6 (95%CI 13.2-18.3) |
| Fisher and Hayden, 2014, British Columbia, Canada | All ages  n=4,609,659 Population of British Columbia | Genetic confirmation n= 481  Strictly clinical n=150  Total n=631 | Cases ascertained from Center for HD, Victoria General Hospital Medical Genetics, Huntington Society of Canada, BC GP and neurologists records, HD family community, BC nursing homes, and DNA diagnostic laboratory  Chart Review was performed | Genetic testing or strictly clinical diagnosis | 2012 | Genetic confirmation:  10.4 per 100,000 (95% CI 9.5-11.4)  Genetic Plus Strictly Clinical:  13.7 per 100,000 (95%  CI: 12.6–14.8) | Genetic confirmation  Total population: 10.4/100,000  (95% CI: 9.5–11.4)  Caucasian: 13.2/100,000 (95% CI 12.0–14.4)  Strictly Clinical  Total population: 3.3 per 100,000 (95% CI: 2.7–3.8)  Caucasian: 4.1 per 100,000 (95% CI 3.4–4.8)  Genetic + strictly clinical:  Total population: 13.7/100,000 (95% CI 12.6-14.8)  Caucasian: 17.2/100,000 (95% CI 15.8-18.6)  By gender (CI not provided)  M=13.8/100,00  F=13.6/100,000  Age adjusted rates (CI not provided)  15-19=0.1/100,000  20-24=0.1/100,000  25-29=0.1/100,000  30-34=0.4/100,000  35-39=0.5/100,000  40-44=1.2/100,000  45-49=1.4/100,000  50-54=1.7/100,000  55-59=1.7/100,000  60-64=1.2/100,000  65-69=0.9/100,000  70-74=1.0/100,000  75-79=0.6/100,000  80-84=0.4/100,000  >85=0.0/100,000 |
| Gavrielov-Yusim, 2021, Israel | Patients receiving care from Maccabi Health Services (MHS) from 2000 to 2019, an insurer and healthcare provider for a quarter of Israel’s population  All adult patients (18 years and older)  1,580,816 MHS members in 2018 | 69 patients with HD were identified in 2018 | Database of MHS. MHS collects diagnoses from hospitals, general practice, specialty clinics, ambulatory clinics, and hospice settings. MHS captures data on medical procedures, imaging, and drug treatments through drug purchases and pharmacy dispensations. All data are linked at the patient level using a unique national patient identifier. | Data were extracted for all adult MHS patients with an HD diagnosis defined by at least one record of ICD-9 code 333.4. All diagnoses included in the study were made by a neurologist or were documented as a chronic disease diagnosis in the patient’s medical record.  Patients diagnosed with drug induced chorea, subacute dyskinesia due to drugs, Sydenham’s chorea or rheumatic chorea after the index HD diagnosis were excluded. | Prevalence was defined as the total number of adult HD cases in 2018. | 4.36 cases per 100,000 (95% CI 3.40-5.52) | Not reported. |
| Gilling, 2017, Denmark | All ages  Total Population not reported | Manifest HD n=1926 | Danish HD Registry database | Clinical or molecular diagnosis | March 1, 2015 | 5-8 per 100 000 | Not Reported |
| Gordon, 2016, US (Navajo territories) | Navajo residents of the Navajo Nation  Population=  275,000 | 0 | Health Service National Patient Information Reporting  System provided the data | Unspecified | July 1, 2006 | 0 | Post-hoc analysis: 57 cases identified among American Indians nationwide  (none was Navajo)  Estimated prevalence 4.1/100,000 |
| Kim, 2015, South Korea | All ages  2009 n=49,773,145  2010 n=50,515,666  2011 n=50,734,284  2012 n=50,948,272  2013 n=51,141,463 | 2009: 222  2010: 182  2011: 171  2012: 181  2013: 197 | Databases of the Rare Diseases Registry and National Health Insurance | For Rare Disease Registry – patients with physician-certified diagnosis  For National Health Insurance - unspecified | July 2009- December 2013 | Based on the National Health Insurance Registry:  0.41/100,000  Based on the Rare Diseases Registry:  0.39 per 100,000 | Based on the National Health Insurance Database  2009=0.45/100,000  2010=0.36/100,000  2011=0.34/100,000  2012=0.36/100,000  2013=0.39/100,000  Based on the Rare Diseases Registry  208/51,141,463  0.41 per 100,000 |
| Kounidas, 2021, North of Scotland | All ages  Total population in 2020: 893,440  Total population in 2016: 893,870 | <134 individuals with manifest HD in 2020  <123 individuals with manifest HD in 2016  (when the number of cases was less than 5, the exact data point was not given to protect anonymity) | Patients identified using NHS Grampian genetic department laboratory and clinic records. Population obtained from published local council demographic data for 2016 and 2019. | 36 or more CAG repeats in *HTT*; considered to have manifest disease in presence of characteristic movement disorder assessed by an experienced European Network of Huntington Disease (EHDN) certified rater. | July 1, 2016 and January 1, 2020 | Prevalence in 2020:  14.6 per 100,000 (95% CI 14.3-15.3)  Prevalence in 2016:  13.5 per 100,000 (95% CI 13.0-14.0) | By location in 2020:  Grampian: 14.5 per 100,000 (95% CI 13.9-15.1)  Highland: 17.8 per 100,000 (95% CI 16.9-18.7)  Island Boards: 4.17 per 100,000 (95% CI 3.0-5.0)  By location in 2016:  Grampian: 12.9 per 100,000 (95% CI 12.2-13.6)  Highland: 17.9 per 100,000 (95% CI 17.0-18.8)  Island Boards: 4.17 per 100,000 (95% CI 3.0-5.0) |
| Morrison, 2011, Northern Ireland | All ages  Total population not reported | 180 | HD register | Genetic testing | 1 January  2001 | 10.6/100,000 | Not Reported |
| Muroni, 2020, Italy (South Sardinia and Cagliari) | All ages  Study carried out in two counties,  South Sardinia n=353,830 Cagliari n=431,955 | 47  South Sardinia=34  Cagliari=13 | One laboratory providing genetic analysis for island of Sardinia, and one tertiary referral center for HD for the island. Additional cases sought from neurologists in the study area and referral centers for patients with HD in Italy. | Molecular Diagnosis  and clinical symptoms of HD | December 31st, 2017 | 5.98/100,000 (95%CI (4.3–7.7)  South Sardinia 9.6/100,000(95% CI 6.4–12.8)  Cagliari 3.0/100,000 (95% CI 1.4-4.6) | Gender  South Sardinia  M=7.4/100,000 (95% CI, 3.4–11.4)  F= 11.2/100,000 (95% CI, 6.6–15.8)  Cagliari  M=: 2.9/100,000 (95% CI, 0.6–5.2)  F= 3.1/100,000 (95% CI, 0.9–5.4) |
| Ohlmeier, 2019, Germany | All ages  Claims data from approximately 4,000,000 insured persons from 70 German statutory health insurances  Total population: 3,325,638  Total male population: 1,649,385  Total female population:  1,676,253 | Total Cases:  308  Total Male Cases: 169  Total Female Cases: 139 | Applied Health Research Berlin Research  Database | At least two outpatient or inpatient ICD-10 codes for HD during the study period were required for case identification | January 2015 to 31 December 2016 | 9.3 per 100,000 (95%-CI: 8.3–10.4) | Gender  Male  10.2/100,000(  95%-CI: 8.8–11.9) Female  8.3/ 100,000 (95%CI: 7.0–9.8)  Age  0-29  0.7/100,000 (95%CI 0.3–1.5)  30-39  4.3/100,000 (95%CI 2.6–7.1)  40-49  10.6/100,000 (95%CI 7.9– 14.0)  50-59  15.0/100,000(95%CI 12.1– 18.5)  60-69  16.8/100,000(95%CI 13.4– 21.0)  70-79  15.0/100,000 (95%CI 11.6– 19.4)  >80  7.7/100,000 (95%CI 4.9– 12.0)  Men/age  0-29=NR  30-39  5.8/100,00(95%CI 3.1– 10.6)  40-49  13.0/100,000 (95%CI 9.1– 18.7)  50-59  16.6 /100,000 (95%CI 12.5– 22.0)  60-69  15.7/100,000 (95%CI 11.3– 21.8)  70-79  17.6 /100,000 (95%CI 12.6– 24.6)  >80  9.3/100,000 (95%CI 5.1–17.2)  Women/age  0-29=NR  30-39  2.9/100,00(95%CI 1.2–6.7)  40-49  8.1 /100,000 (95%CI 5.1– 12.8)  50-59  13.3/100,000 (95%CI 9.7–18.3)  60-69  17.8 /100,000 (95%CI 13.2–24.1)  70-79  12.4 /100,000 (95%CI 8.3–18.5)  >80  6.4/100,000 (95%CI 3.4– 12.1) |
| Panas, 2011, Greece | All ages  Population  n= 10,964,020 | 278 | Neurogenetics Unit, Athens Greece | Molecular diagnosis of HD  UHDRS applied in all cases | January 1995 to December 2008 | 2.5 per 100,000 | Not Reported |
| Roos, 2017, Sweden (Jämtland and Uppsala) | All ages  Jämtland (rural) n=126 765  Uppsala(Urban) n= 348 942 | Jämtland =28  Uppsala= 17 | Electronic medical records in two Swedish counties of Jamtland and Uppsala | Patients with ICD codes HD (G10) and/or dementia in HD (F02.2)  Cases identified through ICD codes were subsequently confirmed through review of medical records | Between 2004 and 2015  Prevalence values in spring 2015 | Jämtland 22.1/100,000  Uppsala= 4.9/100,000 | Not Reported |
| Sackley, 2011, UK | All ages  2004 n= 2,840,202  2005 n=2,948,206  2006 n=2,977,044  2007 n= 2,983,870  2008 n= 2,964,386 | 324 cases identified over the study period | The Health Improvement Network database | Patients were identified as diagnosed with HD if they had a Read code indicating the condition. Read codes indicating a possible diagnosis (other, unspecified chorea) and a family history of the condition were also identified. | December 31, 2004 to 2008 | HD Diagnosis  2004=6.45/100,000  2005=6.51/100,000  2006=6.54/100,000  2007=6.52/100,000  2008=5.96/100,000 | Not Reported |
| Shaw, 2022, Alberta, Canada | Age ≥21 years  Estimated mid-year Alberta adult (≥21 years) population:  2010/11: 2,745, 527  2011/12: 2,739,009  2012/13: 2,862,175  2013/14: 2,947,228  2014/15: 3.027,608  2015/16: 3,070,416  2016/17: 3,104,520  2017/18: 3,139,143  2018/19: 3,183,874  Average 5-yr (2014/15-2018/19): 3,027,608 | 297 prevalent cases in the final year (2018/19)  368 cases over the final 5-year period (2014/15-2018/19) | De-identified province-wide administrative health data released by the Government of Alberta Ministry of Health from the following linkable administrative datasets: Alberta Blue Cross Pharmacy Claims, Alberta Continuing Care Information System, Alberta Precision Laboratories Dataset, Diagnostic Imaging Dataset, Discharge Abstract Database, National Ambulatory Care Reporting System, Pharmaceutical Information Network Dispenses, Population Registry, Practitioner Claims, and Vital Statistics (deaths).  Data from Practitioner Claims, Discharge Abstract Database, and National Ambulatory Care Reporting System datasets requested from April 1, 1997 to facilitate identification of incident & prevalent cases | Cases required 2 diagnostic codes within a two-year span from ICD-9-CM or ICD-10 -CA, appearing in any position in the Discharge Abstract Database or National Ambulatory Care Reporting System. | April 1, 2010 to March 31, 2020 | Average 5-year (2014/15-2018/19) prevalence: 12.15 per 100,000 persons  (95% CI not reported)  2018/19: 9.33 per 100,000 persons  (95% CI not reported) | Prevalence by year (per 100,000 persons) :  2010/11: 7.25  2011/12: 7.59  2012/13: 8.04  2013/14: 8.52  2014/15: 8.75  2015/16: 9.22  2016/17: 9.31  2017/18: 9.40  2018/19: 9.33  95% CI not reported |
| Sipila, 2015, Finland | All ages  Total population not reported | 114 | Finnish Hospital Discharge  Register  Hospital Benchmarking Database | ICD-9 (3334A) and ICD-10 (G10) codes  Patient records were reviewed to confirm diagnosis.  Included patients who had a motor phenotype suggesting HD and a positive genetic test, or patients who had a motor phenotype suggestive of HD and a family history of HD. | 1 January, 1987 and 31 December, 2010 | 2.12/100,000 (95%CI:1.77-2.54) | age-adjusted prevalence of 2.09/100,000 |
| Squitieri, 2016, Italy Molise region | All ages  n=313,341 | 34 | Italian League for Research on Huntington (LIRH) and Neuromed | Genetic testing or clinical diagnosis confirmed using UHDRS | December 2013 | 10.85/100,000 (95% CI: 7.20–14.50) | all mutation carriers, including at-risk  individuals who were positive to the predictive genetic  test  13.40/100,000 (95% CI:9.35–17.45) |
| Squitieri, 2020, Muscat region, Sultanate of Oman | All ages  n=556 731 | 41 cases | National  Genetic Centre of the Royal Hospital in Muscat | Molecular diagnosis  Clinical diagnosis confirmed by direct examination or examination of patient files | August 2013 to March 2019  Prevalence determined 2019 | 7.36 per 100,000 (95% CI 5.40-10.02) |  |
| Sveinsson, 2012, Iceland | All ages  n=311,114 | 3 individuals with HD on prevalence day | Multiple datasources: medical records and discharge diagnoses from all hospitals in Iceland, including records from neurological, psychiatric and genetic departments. Interviews with family members and physicians. | Based on presence of hyperkinetic movement disorder, psychiatric symptoms, progressive cognitive decline, and family history of first degree relative with HD or positive DNA test for HD. | July 1, 2007 | 1.0/100,000  (95%CI 0.2–2.8) | Not Reported |
| Vicente, 2021, Navarre, Spain | 647,554 inhabitants in January 2018 | 1999: 31  2000: 32  2017: 32 | Multiple data sources:  Minimum Basic Data Set at Hospital Discharge (MBDS): all episodes containing the ICD-9-CM code 333.4 until 2015, and the ICD-10-ES (Spanish modification of ICD version 10) code G10 from 2016-2017  Electronic Clinical Records in Primary Care (ECRPC): “Huntington disease, chorea” descriptors  Temporary Work Disability Registry (TWDR): all episodes containing the ICD-9-CM code 333.4 from 2000-2017  Medical Genetics Centre: patients with clinical signs of HD and relatives referred for assessment and testing | Case validation using medical records and clinical assessment by a neurologist and clinical geneticist with HD expertise.  1 of the following inclusion criteria for diagnosis of HD:  1) neurocognitive signs compatible with HD and a genetic test result of >35 CAG repeats in the *HTT* gene  2) neurocognitive signs compatible with HD, without a genetic test result but with a genetically confirmed family history | 1991-2017  Point prevalence reported for December 31 of 1999, 2008, and 2017 | December 31, 1999: 5.70 per 100,000 (95% CI 3.69-7.71)  December 31, 2008: 5.07 per 100,000 (95% CI 3.31-6.83)  December 31, 2017: 4.94 per 100,000 (95% CI 3.23-6.65) | Prevalence on December 31, 2017  Sex :  Men: 4.68 per 100,000 (95% CI 2.31-7.05)  Women : 5.20 per 100,000 (95% CI 2.73-7.67)  Age :  ≤20 : 0 per 100,000  21-40 : 2.64 per 100,000 (95% CI 0.05-5.22)  41-60 : 5.11 per 100,000 (95% CI 1.94-8.27)  >60 : 11.53 per 100,000 (95% CI 6.20-16.85) |

**Supplementary Table 3. Risk of Bias Assessment.**

| Study | Is the study population clearly defined | Were data collection methods standardized? | Were valid clinical criteria (or genetic testing) used to assess for the presence/absence of disease? | Are the estimates of prevalence and incidence given with confidence intervals and in detail by subgroup? | TOTAL QUALITY SCORE |
| --- | --- | --- | --- | --- | --- |
|  |  |  |  |  |  |
| (Agostinho, Da Silva et al. 2015) | 1 | 1 | 1 | 0 | 3 |
| (Baine, Krause et al. 2016) | 1 | 1 | 1 | 0 | 3 |
| (Bruzelius, Scarpa et al. 2019) | 1 | 1 | 0 | 1 | 3 |
| (Carrassi, Pugliatti et al. 2017) | 1 | 1 | 1 | 1 | 4 |
| (Castilhos, Santos et al. 2019) | 1 | 1 | 1 | 0 | 3 |
| (Cubo, Doumbe et al. 2017) | 1 | 1 | 0 | 0 | 2 |
| (Dastgiri, Bonyadi et al. 2012) | 1 | 1 | 1 | 0 | 2 |
| (Demetriou, Heraclides et al. 2018) | 1 | 1 | 1 | 1 | 4 |
| (Douglas, Evans et al. 2013) | 1 | 1 | 0 | 1 | 3 |
| (Evans, Douglas et al. 2013) | 1 | 1 | 0 | 1 | 3 |
| (Fisher and Hayden 2014) | 1 | 1 | 1 | 1 | 4 |
| Gavrielov-Yusim et al, 2021 | 1 | 1 | 0 | 1 | 3 |
| (Gilling, Budtz-Jorgensen et al. 2017) | 1 | 1 | 1 | 0 | 3 |
| (Gordon, Mehal et al. 2016) | 1 | 0 | 0 | 0 | 3 |
| (Kim, Lyoo et al. 2015) | 1 | 1 | 1 | 0 | 3 |
| (Kodra, Minelli et al. 2019) | 1 | 1 | 0 | 0 | 2 |
| (Kounidas, Cruickshank et al., 2021) | 1 | 1 | 1 | 1 | 4 |
| (Morrison, Harding-Lester et al. 2011) | 1 | 1 | 1 | 0 | 3 |
| (Muroni, Murru et al. 2020) | 1 | 1 | 1 | 1 | 4 |
| (Muroni, Murru et al., 2021) | 1 | 1 | 1 | 1 | 4 |
| (Ohlmeier, Saum et al. 2019) | 1 | 1 | 0 | 1 | 3 |
| (Panas, Karadima et al. 2011) | 1 | 1 | 1 | 0 | 3 |
| (Roos, Wiklund et al. 2017) | 1 | 1 | 1 | 0 | 3 |
| (Sackley, Hoppitt et al. 2011) | 1 | 1 | 0 | 0 | 2 |
| (Shaw, Mayer, et al., 2022) | 1 | 1 | 0 | 0 | 2 |
| (Sienes Bailo, Lahoz et al. 2020) | 1 | 1 | 1 | 1 | 4 |
| (Sipila, Hietala et al. 2015) | 1 | 1 | 1 | 1 | 4 |
| (Squitieri, Griguoli et al. 2016) | 1 | 1 | 1 | 1 | 4 |
| (Squitieri, Maffi et al. 2020) | 1 | 1 | 1 | 1 | 4 |
| (Sveinsson, Halldorsson et al. 2012) | 1 | 1 | 1 | 1 | 4 |
| (Vicente, Sabando et al., 2021) | 1 | 1 | 1 | 1 | 4 |
| (Wandell, Fredrikson et al. 2021) | 1 | 1 | 1 | 0 | 3 |
| (Wexler, Collett et al. 2016) | 1 | 1 | 0 | 1 | 3 |

**Supplementary Table 4. Incidence of HD, 1985 to 2022**

| **Location** | **Study** | **Cases** | **Sample (person years)** | **Incidence per 100,000 person-years** | **95% CI** |
| --- | --- | --- | --- | --- | --- |
| Asia (South Korea) | (Kim, Lyoo et al. 2015) | 29 | 51,141,463 | 0.06 | 0.04-0.08 |
| Asia (Taiwan) | Chen, 2010 | 23 | 22,600,000 | 0.10 | 0.06-0.14 |
| ***Subgroup analysis Asia I^2^=0, Q=1*** | | | | **0.08** | **0.03-0.12** |
| Europe (Italy) | Carrasi 2017 | 2 | 2,000,000 | 0.10 | 0.03-0.24 |
| Europe (Italy) | (Kodra, Minelli et al. 2019) | 614 | 180,588,235 | 0.34 | 0.31-0.37 |
| Europe (Italy) | (Muroni, Murro et al, 2021) | 53 | 18,150,685 | 0.29 | 0.21-0.37 |
| Europe (Germany) | (Ohlmeier, Saum et al. 2019) | 60 | 6,651,276 | 0.90 | 0.67-1.13 |
| Europe (Greece) | (Panas, Karadima et al. 2011) | 20 | 9,025,974 | 0.22 | 0.12-0.32 |
| Europe (Spain) | Ramos-Arroyo, 2005 | 12 | 2,553,191 | 0.47 | 0.20-0.74 |
| Europe (UK) | (Sackley, Hoppitt et al. 2011) | 13 | 2,964,386 | 0.44 | 0.20-0.68 |
| Europe (Spain) | (Sienes Bailo, Lahoz et al. 2020) | 4 | 593,387 | 0.67 | 0.01-1.34 |
| Europe (Iceland) | (Sveinsson, Halldorsson et al. 2012) | 8 | 5,714,286 | 0.14 | 0.04-0.24 |
| Europe (UK) | Wexler, Collett et al. 2016) | 199 | 29,522,583 | 0.67 | 0.58-0.77 |
| Europe (Spain) | (Vicente, Ruiz de Sabando et al, 2021) | 63 | 15,750,000 | 0.40 | 0.30-0.50 |
| ***Subgroup analysis Europe I^2^=44, Q=18*** | | | | **0.38** | **0.28-0.49** |
| North America (Canada) | Almqvist, 2001 | 27 | 3,913,044 | 0.69 | 0.43-0.95 |
| North America (U.S.) | (Bruzelius, Scarpa et al. 2019) | 267 | 15,198,207 | 1.76 | 1.55-1.97 |
| North America (Canada) | (Shaw, Mayer et al, 2022) | 21 | 3,183,874 | 0.66 | 0.38-0.94 |
| ***Subgroup analysis North America I^2^=0, Q=2*** | | | | **1.04** | **0.28-1.80** |
| Oceania (Australia) | McCusker, 2000 | 39 | 6,038,696 | 0.65 | 0.44-0.85 |
| ***Pooled estimate I2=63, Q=44*** | | | | **0.47** | **0.36-0.59** |

**Supplementary Table 5. Prevalence of HD 1985 to 2022**

| **Location** | **Study** | **Cases** | **Sample** | **Prevalence per 100,000** | **95% CI** |
| --- | --- | --- | --- | --- | --- |
| Africa (South Africa) | Baine, Krause et al. 201  6 | 384 | 51,489,107 | 0.75 | 0.67-0.82 |
| Africa (Cameroon) | Cubo, Doumbe et al. 2017 | 2 | 3,000,000 | 0.07 | 0.02-0.27 |
| ***Subgroup analysis Africa (Q=1, I^2^=0)*** | | | | **0.25** | **0.02-2.61** |
| Asia (China) | Chang 1994 | 14 | 5,550,000 | 0.25 | 0.15-0.43 |
| Asia (Taiwan) | Chen 2010 | 97 | 23,000,000 | 0.42 | 0.35-0.51 |
| Asia (Israel) | Gavrielov-Yusim, Barer et al, 2021 | 69 | 1,580,816 | 4.36 | 3.45-0.51 |
| Asia (South Korea) | Kim, Lyoo et al. 2015 | 208 | 51,141,463 | 0.41 | 0.36-0.47 |
| Asia (Japan) | Nakashima 1996 | 9 | 1,387,000 | 0.65 | 0.34-1.25 |
| Asia (Sultanate of Oman) | Squitieri, Maffi et al. 2020 | 41 | 556,731 | 7.36 | 5.42-10.00 |
| ***Subgroup analysis Asia (Q=5, I^2^=6)*** | | | | **0.99** | **0.33-2.95** |
| Europe (Italy) | Carrasi 2017 | 15 | 354,673 | 4.23 | 2.55-7.02 |
| Europe (UK) | Evans, Douglas et al. 2013 | 432 | 3,515,986 | 12.29 | 11.18-13.50 |
| Europe (Denmark) | Gilling, Budtz-Jorgensen et al. 2017 | 329 | 5,660,000 | 5.81 | 5.22-6.48 |
| Europe (Wales) | James 1994 | 86 | 1,393,900 | 6.17 | 4.99-7.62 |
| Europe (Scotland) | Kounidas, Cruickshank et al, 2021 | 134 | 893,440 | 15.00 | 12.66-17.77 |
| Europe (Northern Ireland) | Morrison, Harding-Lester et al. 2011 | 180 | 1,698,113 | 10.60 | 9.16-12.27 |
| Europe (Northern Ireland) | Morrison 1994 | 2 | 128,117 | 1.56 | 0.39-6.24 |
| Europe (Italy) | Muroni, Murru et al. 2020 | 47 | 785,785 | 5.98 | 4.49-7.96 |
| Europe (Germany) | Ohlmeier, Saum et al. 2019 | 308 | 3,325,638 | 9.26 | 8.28-10.36 |
| Europe (Greece) | Panas, Karadima et al. 2011 | 278 | 10,964,020 | 2.54 | 2.25-2.85 |
| Europe (Italy) | Pavoni 1990 | 7 | 370,374 | 1.89 | 0.90-3.96 |
| Europe (Slovenia) | Peterlin 2009 | 104 | 2,011,614 | 5.17 | 4.27-6.27 |
| Europe (Sweden) | Roos, Wiklund et al. 2017 (Jamatland) | 28 | 126,765 | 22.09 | 15.25-31.99 |
| Europe (Sweden) | Roos, Wiklund et al. 2017 (Uppsala) | 17 | 348,942 | 4.87 | 3.03-7.84 |
| Europe (UK) | Sackley, Hoppitt et al. 2011 | 177 | 2,964,386 | 5.97 | 5.15-6.92 |
| Europe (UK) | Shiwach 1994 | 138 | 2,437,000 | 5.66 | 4.79-6.69 |
| Europe (Finland) | Sipila, Hietala et al. 2015 | 114 | 5,377,358 | 2.12 | 1.76-2.55 |
| Europe (Italy) | Squitieri, Griguoli et al. 2016 | 34 | 313,341 | 10.85 | 7.75-15.19 |
| Europe (Iceland) | Sveinsson, Halldorsson et al. 2012 | 3 | 311,114 | 0.96 | 0.31-2.99 |
| Europe (Spain) | Vicente, Ruiz de Sabando et al, 2021 | 32 | 647,554 | 4.94 | 3.49-6.99 |
| Europe (UK) | Watt 1993 | 101 | 2,520,000 | 4.01 | 3.30-4.87 |
| ***Subgroup analysis Europe (Q=26, I^2^=22)*** | | | | **5.65** | **4.31-7.41** |
| North America (U.S.) | Bruzelius, Scarpa et al. 2019 | 3707 | 67,582,529 | 5.49 | 5.31-5.66 |
| North America (Canada) | Fisher and Hayden 2014 | 631 | 4,609,659 | 13.69 | 12.66-14.80 |
| North America (U.S.) | Kokmen 1994 | 2 | 106,000 | 1.89 | 0.47-7.54 |
| North America (Canada) | Shaw, Mayer et al, 2022 | 297 | 3,183,874 | 9.33 | 8.33-10.45 |
| ***Subgroup analysis North America (Q=4, I^2^=24)*** | | | | **7.43** | **4.08-13.53** |
| Oceania (Australia) | Mckusker 2000 | 380 | 6,038,696 | 6.29 | 5.69-6.96 |
| Oceania (Australia) | Pridmore 1990 | 54 | 447,000 | 12.08 | 9.25-15.77 |
| *Subgroup analysis Oceania (Q=1, I^2^=0)* | | | | 8.61 | 4.55-16.31 |
| South America (Brazil) | Agostinho, Da Silva et al. 2015 | 13 | 18,087 | 71.87 | 41.74-123.74 |
| South America (Brazil) | Castilhos, Santos et al. 2019 | 209 | 11,297,297 | 1.85 | 1.62-2.12 |
| ***Subgroup analysis South America (Q=1, I^2^=0)*** | | | | **11.42** | **0.32-410.99** |
| ***Total (Q=71, I^2^=50)*** | | | | **3.92** | **2.90-5.30** |

Abbreviations: U.S. = United States; UK= United Kingdom.
